# Ireland

**Published:** 1907-09-07

**Authors:** 


### IRELAND.

*Royal College of Surgeons in Ireland (the Schools of Surgery).*—These schools are attached by charter to the Royal College of Surgeons in Ireland. They are carried on with the college buildings, and are specially subject to the supervision and control of the Council, who are empowered to appoint and remove the professors, and to regulate the methods of teaching pursued. The buildings have been reconstructed, the capacity of the dissecting-room nearly trebled, and special pathological, bacteriological, public health, chemical, and pharmaceutical laboratories fitted with the most approved appliances, in order that students may have the advantage of the most modern methods of instruction. There are special rooms set apart for lady students.

Summer session: Lectures and practical courses commence April 1; terminate June 30.

Winter session: Lectures and practical courses commence October 15; terminate March 31.

The medical students' guide will be forwarded post free on application to the Registrar, Royal College of Surgeons, Dublin.

*Queen's College, Belfast.*—The courses of study meet the requirements of the Royal University of Ireland and other examining bodies.

Clinical instruction is given at the Royal Victoria Hospital and other hospitals.

Full information can be had on application to the Registrar, Queen's College, Belfast.

*Queen's College, Cork.*—Students can attend courses which meet the requirements of the Royal University of Ireland, the Conjoint Boards of London, Edinburgh, or Dublin, and other examining bodies.

Clinical instruction is given at the North and South Infirmaries, each of which contains 100 beds, and at other hospitals.

Further information can be had on application to the Registrar, Queen's College, Cork.

*The Medical School of the Catholic University, Ireland, Dublin.*—Founded in 1855, it is now the largest medical school in Ireland. It prepares students specially for the examinations of the Royal University and the Conjoint Colleges of Ireland and Edinburgh, but its lectures and practical courses are recognised by all the licensing bodies in Great Britain and Ireland. In addition to the ordinary medical examinations it prepares students for the D.P.H. and for the various higher University examinations in pathology, physiology, chemistry, etc. Six exhibitions and numerous gold and silver medals are offered annually for competition. The school opens for dissections on October 1; the winter session begins on November 1.

